# Circulatory Response to Rapid Volume Expansion and Cardiorespiratory Fitness in Fontan Circulation

**DOI:** 10.1007/s00246-021-02802-y

**Published:** 2021-12-17

**Authors:** Thomas Möller, Vibeke Klungerbo, Simone Diab, Henrik Holmstrøm, Elisabeth Edvardsen, Guro Grindheim, Henrik Brun, Erik Thaulow, Alvaro Köhn-Luque, Assami Rösner, Gaute Døhlen

**Affiliations:** 1 Department of Paediatric Cardiology, Oslo University Hospital Rikshospitalet, Nydalen, P.O. Box 4950, 0424 Oslo, Norway; 2 Institute of Clinical Medicine, Faculty of Medicine, University of Oslo, Oslo, Norway; 3 Institute of Physical Performance, Norwegian School of Sport and Sciences, Oslo, Norway; 4 Department of Pulmonary Medicine, Oslo University Hospital Ullevål, Oslo, Norway; 5 Division of Emergencies and Critical Care, Oslo University Hospital - Rikshospitalet, Oslo, Norway; 6 Oslo Centre for Biostatistics and Epidemiology, Faculty of Medicine, University of Oslo, Oslo, Norway; 7 Department of Cardiology, University Hospital of North Norway, Tromsø, Norway

**Keywords:** Fontan circulation, Hemodynamics, Transpulmonary gradient, Ventricular function, Preload challenge, Univentricular congenital heart defects

## Abstract

The role of dysfunction of the single ventricle in Fontan failure is incompletely understood. We aimed to evaluate hemodynamic responses to preload increase in Fontan circulation, to determine whether circulatory limitations in different locations identified by experimental preload increase are associated with cardiorespiratory fitness (CRF), and to assess the impact of left versus right ventricular morphology. In 38 consecutive patients (median age = 16.6 years, 16 females), heart catheterization was supplemented with a rapid 5-mL/kg body weight volume expansion. Central venous pressure (CVP), ventricular end-diastolic pressure (VEDP), and peak systolic pressure were averaged for 15‒30 s, 45‒120 s, and 4‒6 min (steady state), respectively. CRF was assessed by peak oxygen consumption (VO_2peak_) and ventilatory threshold (VT). Median CVP increased from 13 mmHg at baseline to 14.5 mmHg (*p* < 0.001) at steady state. CVP increased by more than 20% in eight patients. Median VEDP increased from 10 mmHg at baseline to 11.5 mmHg (*p* < 0.001). Ten patients had elevated VEDP at steady state, and in 21, VEDP increased more than 20%. The transpulmonary pressure difference (CVP‒VEDP) and CVP were consistently higher in patients with right ventricular morphology across repeated measurements. CVP at any stage was associated with VO_2peak_ and VT. VEDP after volume expansion was associated with VT. Preload challenge demonstrates the limitations beyond baseline measurements. Elevation of both CVP and VEDP are associated with impaired CRF. Transpulmonary flow limitation was more pronounced in right ventricular morphology. Ventricular dysfunction may contribute to functional impairment after Fontan operation in young adulthood.

*ClinicalTrials.gov* *identifier* NCT02378857

## Introduction

Palliative operations for univentricular congenital heart defects are among the most frequently performed procedures in pediatric open-heart surgery. Fontan-type palliation has saved many lives during the last four to five decades [1]. However, with few exceptions, patients live with major limitations in cardiorespiratory fitness (CRF) [2], and the ability to improve performance and predict the post-Fontan clinical course is disappointingly poor.

Long-term failure of the low-energy/low-flow Fontan circulation is inevitable [3]. During diagnostic right heart catheterization, elevation of the central venous pressure (CVP), often called Fontan pressure, is the most informative functional variable. Hence, chronically elevated pulmonary vascular resistance has been considered the key to understanding and treating Fontan failure. Pulmonary vasodilators have failed to achieve substantial improvement in cardiorespiratory fitness (CRF) or any improvement in long-term survival [4]. The second potential cause of elevated CVP, i.e., failure of the single ventricle and increased filling pressure, has received less investigative attention.

Sudden increase in preload during right heart catheterization has previously been used by other groups to evaluate the hemodynamic changes and limitations of the Fontan circulation [5, 6]. However, these previous experiments did not evaluate whether hemodynamic limitations correspond with impaired CRF, which is critical for functional status and patient prognosis [7–9]. This study aimed to (1) characterize the extent and level of hemodynamic responses to acute increase in preload following rapid saline infusion in a representative sample of adolescent patients with Fontan circulation, (2) investigate whether hemodynamic limitation correlated with impaired CRF, and (3) verify whether hemodynamic and functional responses (and limitations) were different between the left and right ventricular morphologies.

## Methods

### Design and Study Population

The present experimental study was part of the Norwegian Fontan Project at Oslo University Hospital, which is a multi-disciplinary observational study involving a national cohort of adolescents living with Fontan circulation.

All patients were recruited between March 2015 and December 2018 during routine clinical work-up before transition to adult care. In Norway, which has 5 million inhabitants, Oslo University Hospital is the only surgical center that performs cardiac surgery and catheter-based interventions in patients of all ages with congenital heart disease. Transition to adult health care usually occurs at 18 years of age. During the last 2 years prior to transition, we routinely admitted patients with Fontan circulation for a comprehensive diagnostic work-up. On two separate days of admission, all patients underwent heart catheterization and a cardiopulmonary exercise test (CPET). The inclusion criterion for the present study was pre-transitional hospital admission. Patients with other severe sensory or neurodevelopmental health problems, for whom the expected diagnostic gain was considered small when compared with the procedural burden, were excluded. Heart catheterization was performed in all enrolled patients unless they had recently undergone a clinically indicated catheterization.

### Heart Catheterization Procedure, Preload Challenge, and Hemodynamic Assessment

Heart catheterization was performed under either conscious sedation or general anesthesia depending on the patient’s request or feasibility. The patients were prepared for the procedure by an initial infusion of 5 mL of 0.9% saline solution/kg body weight which equals 50% of the preprocedural volume support in Fontan patients per institutional protocol. Femoral arterial and venous access were used. Hemodynamic assessments were performed as triplet pressure measurements with liquid-filled catheters after blood sampling from each of the following locations: descending aorta, ascending aorta, ventricular cavity, superior vena cava, right pulmonary artery, left pulmonary artery, inferior vena cava, bilateral pulmonary wedge position, and hepatic venous wedge position. All pressure readings were obtained at the end of expiration, from maximum pressures in patients with spontaneous breathing and from minimum pressures in ventilated patients.

For the preload challenge, we performed rapid volume expansion (RVE) manually by rapidly (over 15‒30 s) infusing 5 mL of 0.9% saline solution/kg body weight at room temperature. Saline was simultaneously infused through at least two venous access sites.

Serial pressure readings (mmHg) were recorded simultaneously at precise time intervals after the onset of saline infusion: every 15 s for the first 2 min, every 30 s for the next 2 min, and every minute until 6 min after infusion. Pressure readings were averaged for 15‒30 s, 45‒120 s, and 4‒6 min (steady state), and the maximum pressure at any stage after baseline was identified. Pressure measurements before and after RVE were obtained from the ventricular cavity in systole (VSP) and end-diastole (VEDP), the inferior vena cava (CVP), and the hepatic wedge position. The difference between CVP and VEDP (CVP‒VEDP) was calculated and considered as indicative of, but not equal to, the transpulmonary pressure gradient.

There is no generally accepted definition of the limits of normal pressure in Fontan circulation. Based on clinical experience and published invasive pediatric data [10, 11], we considered individual pressure readings as elevated if the CVP ≥ 18 mmHg, VEDP ≥ 15 mmHg, or CVP‒VEDP ≥ 6 mmHg. Pressure curve analyses were performed offline using the Axiom Sensis XP angiographic lab system (Siemens Healthcare, Erlangen, Germany).

### Cardiopulmonary Exercise Test

All patients performed a maximal symptom-limited CPET on a treadmill (Woodway, Weil am Rhein, Germany) using the Oslo test protocol [12]—the same protocol used for the reference population to calculate the predicted peak oxygen consumption (VO_2peak_) [13]. The patients breathed into a two-way breathing mask (7450 series, Hans Rudolph Inc., Shawnee, KS, USA), where gas exchange and ventilatory variables were directly determined by breath-by-breath sampling and averaged over 30-s intervals (MasterScreen CPX Metabolic Cart, Jaeger, Hoechberg, Germany). The peak heart rate was measured using a 12-lead electrocardiograph (Custo Cardio 100, CustoMed, Ottobrunn, Germany). All tests were performed by an experienced physiotherapist or exercise physiologist in the presence of a physician, and all patients were familiarized with treadmill running. Prior to each test, the metabolic cart was calibrated for volume and gas, according to the manufacturer’s standards.

The primary outcome during CPET was CRF, expressed as VO_2peak_ [mL × kg^−1^ × min^−1^], and oxygen consumption at the ventilatory threshold (VO_2_@VT). VT was calculated using a combined method with the ventilatory equivalent and the V-slope method [14] to assess concurrent break points and to eliminate false breakpoints, and expressed as a percentage of the measured VO_2peak_. We chose VO_2peak_ due to its prognostic impact and VO_2_@VT because it reflects the circulatory (aerobic) component of the total functional reserve of the body [2, 7].

### Ethical Considerations

All study participants provided informed consent before enrollment. The study protocol was approved by the Regional Committee for Medical and Health Research Ethics (REK Sør-Øst, file no. 2013/1331) and registered with ClinicalTrial.gov (identifier: NCT02378857).

### Statistical Analyses

Continuous variables were presented as mean ± standard deviation for normal distribution; otherwise, median/range and interquartile range (IQR) were provided. Comparisons were made using paired or non-paired two-tailed Student’s *t*-test for normal distribution and otherwise by Mann–Whitney *U* test or one-way repeated-measures analysis of variance (ANOVA) as appropriate for the number of groups and variables. Normality tests were performed using the Shapiro–Wilk test. Variable relationships were analyzed with univariate and multivariate linear regression analyses.

To analyze group differences and account for the individual variation across the repeated measurements (baseline, 45‒120 s, and 4‒6 min), we applied linear mixed-effect models. Specifically, we accounted for individual variability by unique random intercepts for each patient. To adjust for the impact of ventricle type and/or sedation type on the measurements, ventricle type and/or sedation type were included as a fixed effect. Model parameters and confidence intervals were estimated using the restricted maximum likelihood estimation. Model selection was done using the Schwarz’s Bayesian criterion. Statistical significance was set at *p* < 0.05. Statistical analysis was performed using IBM Statistical Product and Service Solutions for Windows (SPSS), versions 26.0 and 27.0 (IBM Corp., Armonk, NY, USA).

### Patient and Public Involvement

The Norwegian Association for Children with Congenital Heart Disease was involved in designing the study and planning organization of the enrollment phase. The sequence of study tests was revised based upon invited feedback from participants. A representative of the Norwegian Association for Adults with Congenital Heart Disease participated in continuous safety surveillance with the study monitoring group.

## Results

**Fig. 1:**
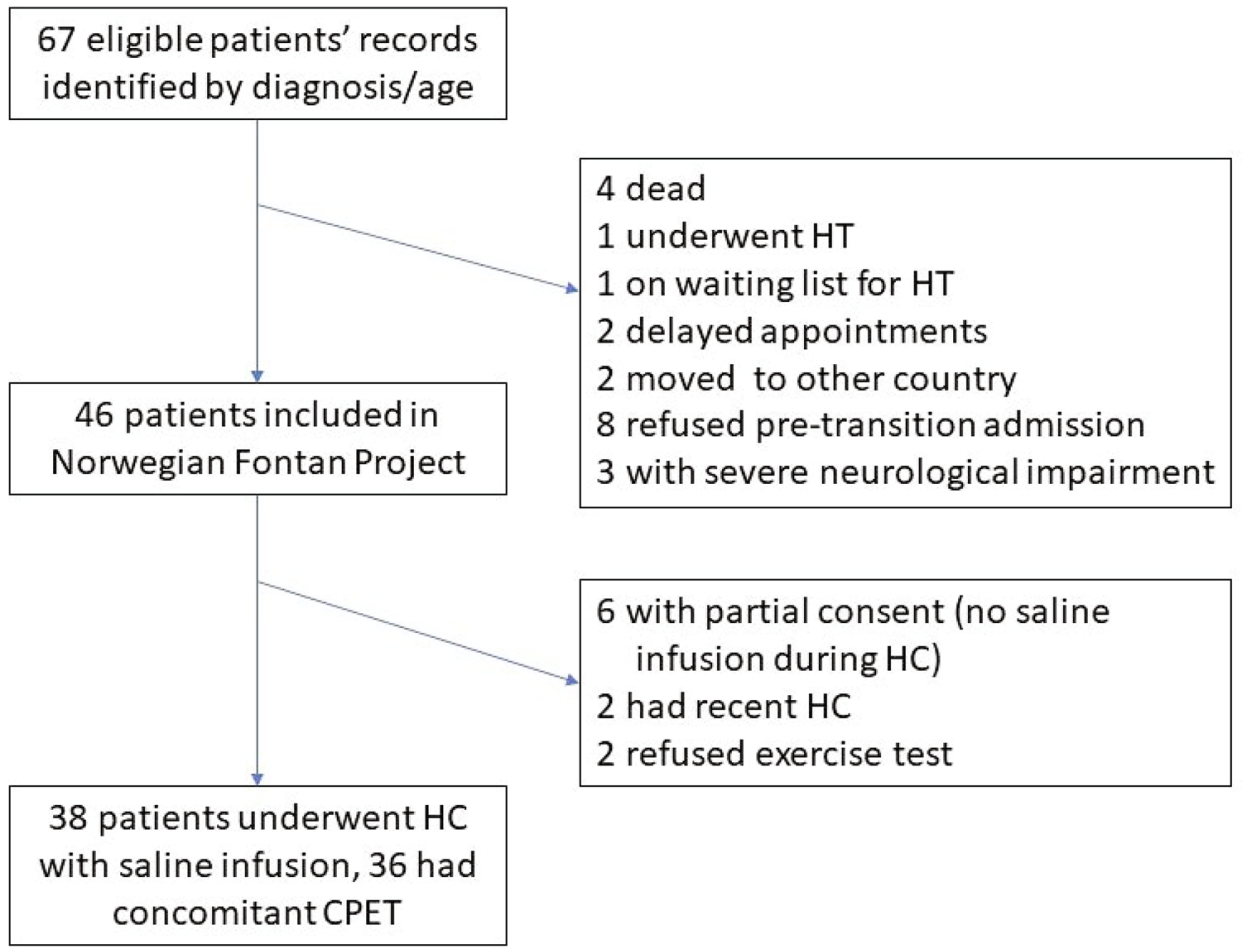
Flowchart of patient enrollment in the present study. *CPET* cardiopulmonary exercise test, *HC* heart catheterization, *HT* heart transplantation

During the study period, we included 38 patients who gave their informed consent to undergo experimental intravenous saline infusion during routine catheterization. The inclusion and general characteristics of the study population are shown in Fig. 1 and Table 1. Heart catheterization was performed under conscious sedation in 16 patients and general anesthesia in 22 patients (14 intubated and 8 with laryngeal mask). Seven patients had pacemakers and were selectively excluded from the heart rate variation analysis.

**Table 1:** General characteristics of the study population with heart catheterization and preload challenge (*n* = 38)

Variable	Value	%
Median age [years]	16.6. (15.4–17.9)	
Sex [female/male]	16/22	42/58
Median body mass index [kg/m^2^]	20.9 (15.3–31.0)	
Median oxygen saturation at rest [%]	95 (91–98)	
Median age at Fontan operation [years]	2.0 (1.0–11.0)	
Median elapsed time since Fontan operation [years]	14.4 (6.0–16.7)	
Fontan-type (extra-cardiac/lateral tunnel)	28/10	74/26
Systemic ventricular morphology (LV/RV/common)	19/16/3	50/42/8
*Echocardiographic features at inclusion*		
Atrioventricular valve regurgitation (none/mild/moderate)	11/22/5	29/58/13
Open fenestration	1	
Protein-losing enteropathy (by clinical judgment)	4	11
*Anatomic diagnoses*		
Hypoplastic left heart syndrome	10	26
Tricuspid atresia	8	21
Double outlet right ventricle	4	11
Double inlet left ventricle	4	11
Pulmonary atresia/intact ventricular septum	3	8
Other (unbalanced AVSD, hypoplastic right ventricle, etc.)	9	23
Heterotaxy syndrome	1	3
*Devices*		
Pacemaker	6	16
ICD	0	
*Medication*		
Acetylsalicylic acid	26	68
Warfarin	10	26
Angiotensin-converting enzyme inhibitor	6	16
Pulmonary vasodilator (sildenafil and/or bosentan)	4	11
Diuretics (furosemide and/or hydrochlorothiazide)	5	13
Aldosterone antagonist	3	8
Beta-blocker	2	5
Antiarrhythmic drugs (non-beta-blocker)	0	

*LV/RV* left/right ventricular morphology, *AVSD* atrioventricular septal defect, *ICD* implantable cardioverter-defibrillator

Values are counts (*n*) unless otherwise specified

Data from complete hemodynamic assessment at baseline and after RVE, including group differences, are provided in Table 2.

**Table 2:** Hemodynamic parameters at baseline and after rapid volume expansion (RVE)

Heart rate (non-pacemaker patients) [beats per minute]	All (*N* = 30)	*p*-value vs. baseline	LV (*N* = 13)	RV (*N* = 14)	Self-breathing (*N* = 13)	General anesthesia (*N* = 18)
Baseline	75.5 ± 15.3		73.9 ± 16.8	75.7 ± 15.4	69.8 ± 16.1	79.0 ± 13.7
Lowest during the 1st minute after RVE	65.9 ± 13.8	< 0.001	62.5 ± 12.8	68.2 ± 15.8	61.9 ± 13.5	68.6 ± 13.4
Steady state	72.4 ± 14.2	< 0.001	68.7 ± 14.8	74.2 ± 14.5	66.9 ± 14.8	75.9 ± 12.5

Pressure measurements [mmHg]	All (*N* = 38)	*p*-value vs. baseline	LV (*N* = 19)	RV (*N* = 16)	(*N* = 16)	(*N* = 22)
*Hepatic wedge pressure*						
Baseline	14.4 ± 3.5		13.4 ± 3.4	15.6 ± 3.0	13.3 ± 3.5	15.2 ± 3.4
Maximum after RVE	18.1 ± 3.7	< 0.001	17.3 ± 3.9*	19.1 ± 3.0	17.2 ± 4.0	18.7 ± 3.5
Average 45‒120 s after RVE	16.9 ± 3.4	< 0.001	16.0 ± 3.5	18.0 ± 2.7	16.2 ± 3.5	17.3 ± 3.3
Steady state	15.6 ± 3.3	< 0.001	14.6 ± 3.2*	17.0 ± 2.7*	14.7 ± 3.3	16.3 ± 3.2
*Central venous pressure (CVP)*						
Baseline	13.2 ± 3.4		12.3 ± 3.3	14.6 ± 2.9	12.0 ± 3.6	14.1 ± 3.0
Maximum after RVE	16.7 ± 3.4	< 0.001	15.7 ± 3.7*	18.1 ± 2.2	15.8 ± 3.9	17.4 ± 2.9
Average 45‒120 s after RVE	15.7 ± 3.1	< 0.001	14.9 ± 3.4	16.9 ± 2.1	15.3 ± 3.6	16.2 ± 2.7
Steady state	14.6 ± 3.1	< 0.001	13.5 ± 3.0*	16.1 ± 2.2	13.6 ± 3.1	15.3 ± 2.9
*Ventricular end-diastolic pressure (VEDP)*						
Baseline	10.7 ± 4.2*		10.7 ± 4.6	10.7 ± 3.2	11.2 ± 4.7	10.3 ± 3.8
Maximum after RVE	15.2 ± 5.2*	< 0.001	15.3 ± 5.0	14.6 ± 3.7	15.8 ± 5.3	14.7 ± 5.2
Average 45‒120 s after RVE	13.4 ± 4.2	< 0.001	13.7 ± 4.4	12.9 ± 3.5	14.3 ± 4.8	12.7 ± 3.6*
Steady state	12.5 ± 3.9	< 0.001	12.6 ± 19.5	12.7 ± 3.3	13.4 ± 4.5	11.9 ± 3.4
*CVP‒VEDP*						
Baseline	2.6 ± 3.4		1.5 ± 3.0	3.9 ± 3.6	0.8 ± 2.7	3.8 ± 3.3
Maximum after RVE	3.9 ± 3.1	< 0.001	2.7 ± 2.4	5.4 ± 3.6	2.3 ± 3.3	5.1 ± 2.4
Average 45‒120 s after RVE	2.3 ± 3.4	NS	1.2 ± 2.8	4.0 ± 3.5	1.1 ± 3.3	3.5 ± 2.9
Steady state	2.2 ± 3.3	NS	1.0 ± 3.2	3.4 ± 3.2	0.3 ± 3.1	3.6 ± 2.7
*Ventricular peak systolic pressure*						
Baseline	95.4 ± 15.4*		99.1 ± 18.9*	92.5 ± 10.0	101.8 ± 19.4	90.8 ± 9.9
Maximum after RVE	102.0 ± 15.9*	< 0.001	105.6 ± 19.7*	98.9 ± 10.2	108.3 ± 19.9	97.4 ± 10.6
Average 45‒120 s after RVE	98.5 ± 14.7	< 0.001	101.4 ± 18.2	96.0 ± 9.9	104.6 ± 17.9*	94.0 ± 10.1
Steady state	96.9 ± 14.8*	0.025	99.4 ± 18.5*	95.2 ± 9.8*	102.9 ± 17.7	92.6 ± 10.6

*LV/RV* left/right ventricular morphology, *CVP* central venous pressure, *s* seconds, *VEDP* ventricular end-diastolic pressure

Normal distribution of data confirmed with the Shapiro–Wilk test unless marked by *. Comparisons performed as appropriate with paired/non-paired Student’s *t*-test, related samples Wilcoxon signed rank test, or Mann–Whitney *U* test

### Heart Rate Response

**Fig. 2:**
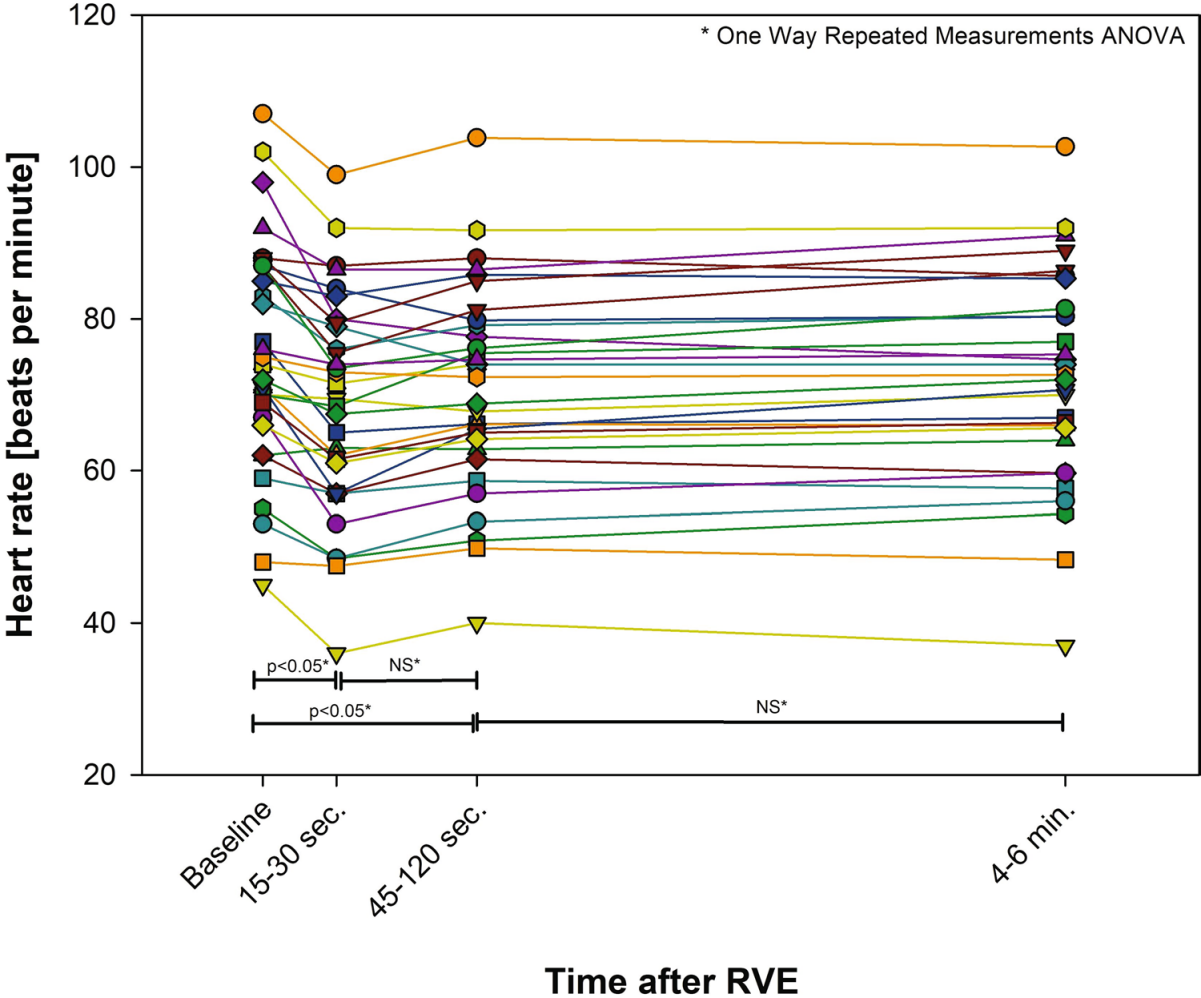
Heart rate response to rapid volume expansion (RVE) in non-pacemaker patients (*N* = 31)

Heart rate reduced significantly in 30 of the 31 non-pacemaker patients (97%) within the first minute after RVE (Fig. 2).

### Median Central Venous Pressure

**Fig. 3:**
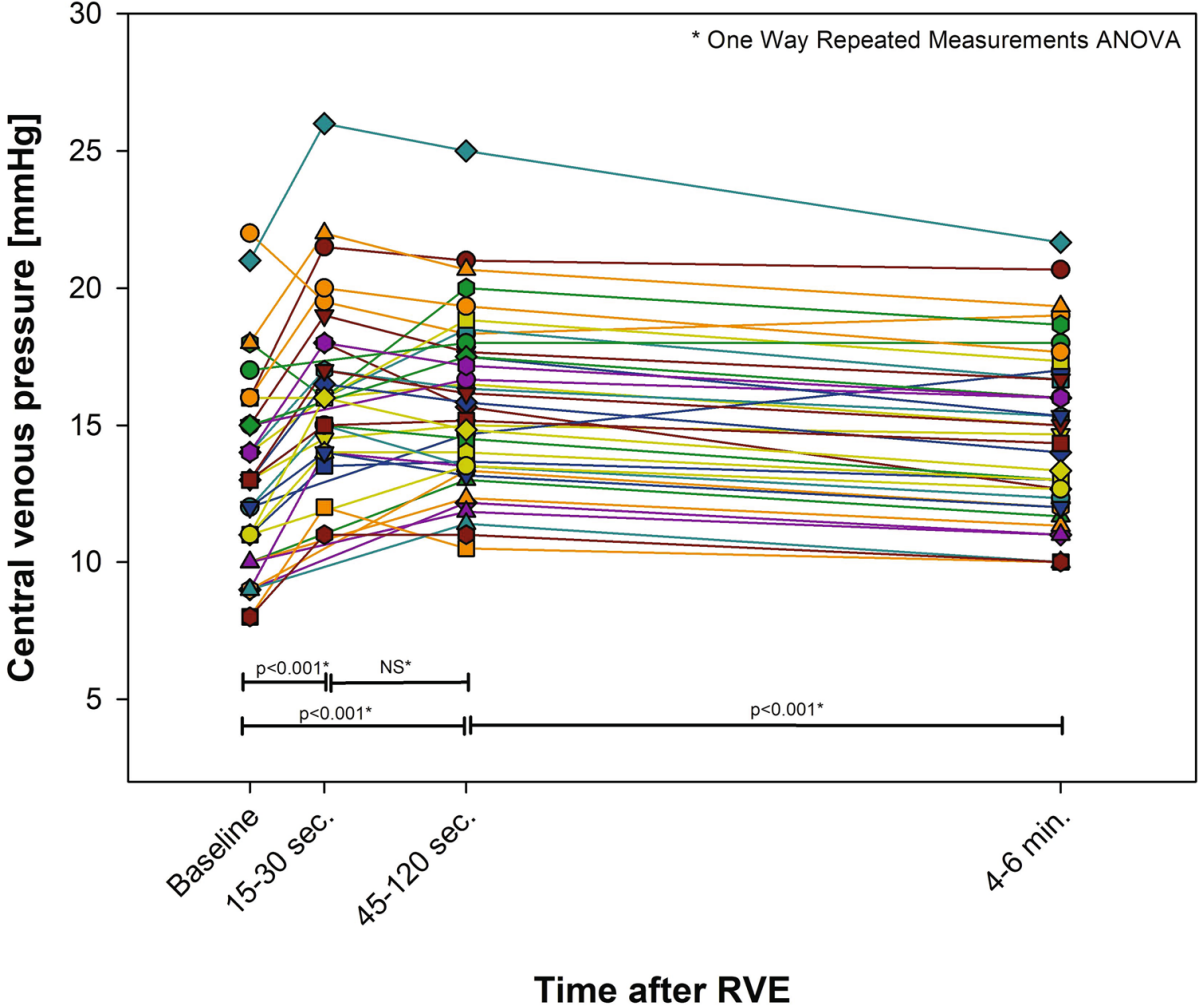
Central venous pressure before and after rapid volume expansion (RVE)

Eight patients (21%) had > 20% increase in CVP after RVE. Four patients had elevated CVP at both baseline and steady state, and in two additional patients, CVP became elevated at steady state (Fig. 3). There was no difference in CVP at any stage between the self-breathing and anesthetized patients.

### Ventricular End-Diastolic Pressure

**Fig. 4:**
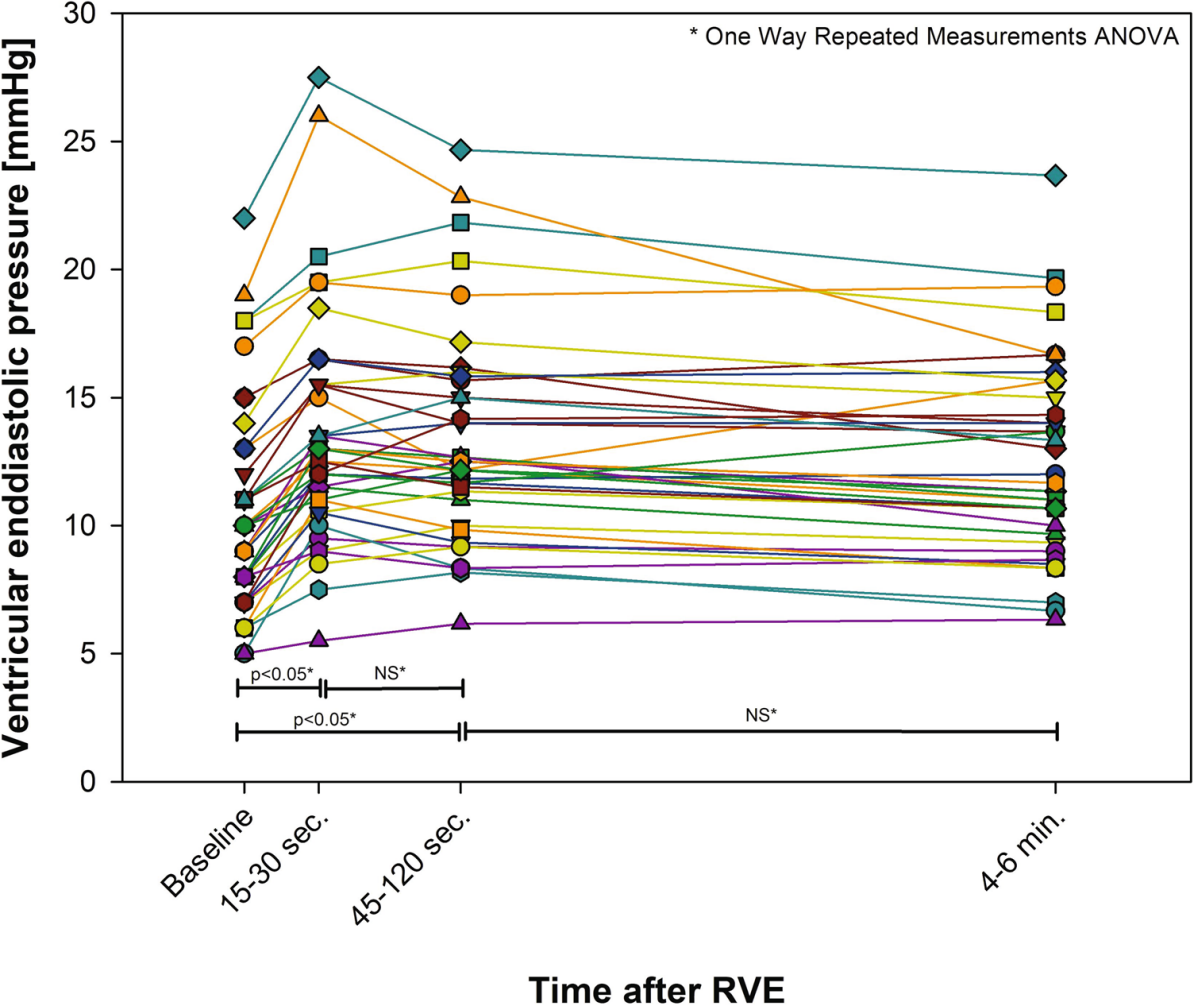
Ventricular end-diastolic pressure before and after rapid volume expansion (RVE). Central illustration: Rapid volume expansion by intravenous saline bolus unmasks limitations of the Fontan circulation by pressure rise upstream from the blood flow restriction(s)

Twenty-one patients (55%) had > 20% increase in VEDP after RVE. Seven patients had elevated VEDP levels at the baseline. Six of these and four additional patients had elevated VEDP at steady state (Fig. 4). There was no difference in VEDP at any stage between the self-breathing and anesthetized patients.

### Pressure Difference Across the Pulmonary Vascular Bed

Nine patients had elevated CVP–VEDP at baseline, of which four were still elevated at steady state. There were no differences between stages detectable by one-way repeated-measures ANOVA. Self-breathing patients had lower CVP–VEDP than anesthetized patients at baseline (0.8 ± 2.7 vs. 3.8 ± 3.3, *p* = 0.004), at 45‒120 s after RVE (0.8 ± 3.4, vs. 3.5 ± 2.9, *p* = 0.014), and at steady state (0.3 ± 3.1, 3.6 ± 2.7, *p* = 0.002).

### Ventricular Peak Systolic Pressure

There was a trend toward approximately 10 mmHg higher VSP in self-breathing patients than in anesthetized patients, and this difference reached statistical significance at steady state (baseline 101.8 ± 19.4 vs. 90.8 ± 9.9 mmHg, maximum 108.3 ± 19.9 vs. 97.4 ± 10.6 mmHg, steady state 102.9 ± 17.7 vs. 97.4 ± 10.6 mmHg, *p* = 0.049).

### The Relationship Between Central Venous Pressure and Ventricular End-Diastolic Pressure

Only 2/7 patients with elevated VEDP at baseline also had elevated CVP at baseline. Only 2/21 patients with preload-induced increase in VEDP > 20% (baseline to steady state) had abnormal CVP at baseline.

Of the nine patients with elevated CVP–VEDP at baseline, only two had elevated CVP. Of 11 patients with elevated CVP–VEDP after RVE, only two had elevated CVP at baseline. Only 2/6 patients had elevated CVP and CVP–VEDP but normal VEDP at steady state after RVE; in contrast, 6/10 patients with elevated VEDP at steady state after RVE did not have simultaneously elevated CVP–VEDP or CVP.

### Cardiopulmonary Exercise Test

Cardiopulmonary exercise data were available for 36 out of the 38 study patients, as one patient refused CPET due to fatigue, and another refused to use a face mask during CPET. In one patient, VT could not be determined.

Thirty-six patients underwent CPET with an exercise test duration until termination of 9.5 ± 2.4 min. The median respiratory exchange ratio at test termination was 1.15 (1.10‒1.20). The maximum heart rate was 177 ± 17.0 beats per minute. VO_2peak_ and VO_2_@VT, including comparison of results for LV and RV morphology, are displayed in Table 3.

**Table 3:** Results from the cardiopulmonary exercise testing

	N	All	LV (*N* = 19/18)	RV (*N* = 15)	*p*-value (LV vs. RV)
VO_2peak_ [mL × kg^−1^ × min^−1^]	36	31.6 ± 7.6	32.9 ± 7.0	29.6 ± 7.9	0.354*
VO_2peak_ [percent of predicted]	36	59 ± 13	62 ± 13	55 ± 12	0.087
VO_2_ at VT [mL × kg^−1^ × min^−1^]	35	21.6 ± 4.6	22.5 ± 3.8	20.8 ± 5.4	0.302
VO_2_ at VT [% of VO_2peak_]	35	70 ± 8.7	70 ± 8.6	71 ± 9.0	0.746
VO_2_ at VT [% of predicted]	35	44 ± 7.9	43 ± 7.0	39 ± 8.7	0.116

*VO*_2_ oxygen consumption, *VO*_2peak_ peak oxygen consumption, *VT* ventilatory threshold, *LV/RV* left/right ventricular morphology

*Mann–Whitney *U* test, other non-paired two-tailed Student’s *t*-test

### Associations Between Hemodynamic Response and Cardiorespiratory Fitness

Univariate analysis showed a significant but low association between VO_2peak_ and CVP at all stages, and no association between VEDP or CVP‒VEDP and VO_2peak_. For VO_2_@VT, the same association was shown for CVP at all stages. Similarly, VEDP at all stages was associated with VO_2_@VT, whereas CVP‒VEDP was not (Table 4).

**Table 4:** Associations of hemodynamic variables with cardiorespiratory fitness by linear regression analysis

Pressure readings at baseline and after RVE [mmHg]	VO_2peak_ [mL × kg^−1^ × min^−1^]				VO_2_@VT [% of VO_2peak_]			
	Univariate				Univariate			
	*β*	95% CI	*R*^2^	*p*	*β*	95% CI	*R*^2^	*p*
CVP baseline	− 0.84	− 1.56 to − 0.12	0.143	0.023	1.67	0.72‒2.65	0.277	0.001
CVP 45‒120 s (average)	− 0.99	− 1.75 to − 0.22	0.169	0.013	1.80	0.83‒2.77	0.302	0.001
CVP maximum	− 0.90	− 1.61 to − 0.19	0.162	0.015	1.72	0.85‒2.58	0.332	< 0.001
CVP at 4‒6 min (average)	− 0.99	− 1.78 to − 0.20	0.161	0.015	1.68	0.69‒2.66	0.267	0.001
VEDP baseline				0.26	1.03	0.23‒1.83	0.173	0.013
VEDP 45‒120 s (average)				0.508	0.96	0.15‒1.77	0.15	0.022
VEDP maximum				0.48	0.77	0.03‒1.52	0.119	0.043
VEDP at 4‒6 min (average)				0.368	0.90	0.06‒1.74	0.127	0.036
CVP‒VEDP baseline				0.51				0.953
CVP‒VEDP 45‒120 s (average)				0.144				0.844
CVP–VEDP maximum				0.189				0.909
CVP‒VEDP at 4‒6 min (average)				0.362				0.848

*CVP* central venous pressure, *min* minutes, *VEDP* ventricular end-diastolic pressure, *RVE* rapid volume expansion, *s* seconds, *VO*_*2peak*_ peak oxygen consumption, *VO*_*2*_*@VT* ventilatory threshold at VO_2peak_

Multivariate linear regression was performed to determine associations between VO_2peak_ or VO_2_@VT and CVP at baseline, VEDP at 45‒120 s, or CVP‒VEDP at 45‒120 s. No significant correlations were found for the combination of the three independent variables or combination of only CVP at baseline and VEDP at 45‒120 s.

Separate univariate analysis for patients with LV morphology did not reveal an association between VO_2peak_ or VO_2_@VT and measured values or percentage changes of CVP, VEDP, or CVP‒VEDP at any stage. However, for RV morphology, the same set of univariate analyses showed an association between VO_2_@VT and CVP at 45‒120 s (*F*(1,13) = 5.514, *p* = 0.035, *R*^2^ = 0.298), maximum CVP (*F*(1,13) = 10.672, *p* = 0.006, *R*^2^ = 0.451), and CVP at steady state (*F*(1,13) = 5.929, *p* = 0.03, *R*^2^ = 0.313), but not for baseline CVP.

### Differences Between Left and Right Ventricular Morphology

Mixed-effect model analyses comparing groups with different ventricular morphologies revealed that patients with right ventricular morphology have, on average, 2.30 mmHg higher CVP (0.36–4.24, *p* = 0.022) and 2.53 mmHg higher CVP‒VEDP (0.39–4.66, *p* = 0.021) than patients with left ventricular morphology. No significant differences between the two morphologies were found for VEDP (− 2.96 to 2.41, *p* = 0.839), ventricular systolic pressure (− 15.75 to 4.98, *p* = 0.299), and heart rate (non-pacemaker patients) (− 8.79 to 14.56, *p* = 0.616).

### Differences Between Self-breathing and Anesthetized Patients

Mixed-effect model analyses comparing patient groups with different sedation showed that ventilated patients have, on average, 3.28 mmHg higher CVP-VEDP than self-breathing patients (1.28–5.28, *p* = 0.002). This effect was still significant having controlled for ventricular morphology (1.05–4.88, *p* = 0.003). Conversely, no significant differences between the different sedation groups were found for CVP (− 1.26 to 3.87, *p* = 0.065), ventricular systolic pressure (− 19.80 to 0.35, *p* = 0.058), and heart rate (non-pacemaker patients) (− 3.42 to 19.71, *p* = 0.160).

## Discussion

In this study, we aimed to characterize hemodynamic responses to acute preload increase by rapid saline infusion in a representative sample of adolescent patients with Fontan circulation. Our volume expansion experiment demonstrated cardiac and circulatory responses to a sudden preload increase, and thereby challenges the reserves in terms of transpulmonary blood flow and functional reserve in a single ventricle. We were able to identify characteristics of preload response and to unmask circulatory limitations that were not detected by baseline pressure assessment.

### Heart Rate Response

We observed a uniform decrease in heart rate immediately after preload increase, which is counterintuitive and contradicts the normal volume-induced increase in heart rate in biventricular physiology [15]. To our knowledge, this phenomenon has not been described in the Fontan circulation before. A potential explanation for immediate heart rate depression is the cooling effect of a fluid bolus at room temperature, as previously described by Wall et al. [16]. However, during the first two minutes of their experimental study, no heart rate differences between warm a cold fluid bolus was observed. Therefore, in the absence of neurophysiological data from our experiment, we hypothesize that the uniform and immediate heart rate reduction was caused by the arterial baroreceptor reflex, which antagonizes the better-known Bainbridge reflex [17]. As the Bainbridge reflex is triggered by receptors in the venous system (i.e., vena cava and right atrium), chronic venous hypertension in Fontan circulation might neutralize this particular reflex circuit.

### Central Venous Pressure Response

Many patients had a significant increase in CVP after RVE, which on a group level is in line with the physiological increase of CVP in the biventricular circulation in young adults [15]. Our data indicate that CVP at baseline does not correlate with elevated VEDP at baseline, and it does not predict an abnormal rise in VEDP or CVP‒VEDP after preload increase. Despite this apparent inability of baseline CVP to indicate limited reserves in the pulmonary vessels or ventricular function, CVP measurement serves as an overall marker of function in Fontan circulation, which is confirmed by its association with VO_2peak_ and VO_2_@VT. However, CVP alone does not permit conclusions about the location of downstream restrictions, either in the pulmonary vessels or in the single ventricle.

### Ventricular Filling Pressure Response

Baseline VEDP was comparable with the reference pressure conditions in young adults [15]. Most of the CVP-responsive patients (> 20% increase) had a simultaneous increase in VEDP without signs of increasing CVP–VEDP, indicating limitations in ventricular function rather than limitations in pulmonary vascular distensibility. These findings challenge the ruling paradigm that pulmonary vascular resistance is the main limiting factor of cardiac output in Fontan circulation [18].

### Changes in Transpulmonary Pressure Difference

Despite preload challenge and increased transpulmonary blood flow, CVP‒VEDP was remarkably stable, and it was not associated with CRF, which contributes to the above-mentioned paradigm challenge. The lack of association with CRF might explain the general disappointing effect of pulmonary vasodilators on CRF [19, 20]. The increase of CVP‒VEDP by positive pressure ventilation during heart catheterization reminds us of how conclusions must be carefully drawn, even from invasive hemodynamic measurements.

### Impact of Ventricular Morphology

We found unfavorable hemodynamic conditions in our subgroup with single RV, compared with single LV. The RV patients had higher pressures in the Fontan circuit at baseline, maximum pressure, and steady state after RVE. Not surprisingly, we found a non-significant trend toward lower CRF in patients with RV than in those with LV morphology, as expected from previous data [2, 21]. It is remarkable that the hemodynamic differences between LV and RV, representing the bottleneck of Fontan circulation, manifest further upstream by elevated CVP‒VEDP and not primarily by elevated VEDP. The common presence of atrioventricular valve insufficiency in single RV may play a role here. Other possible explanations may be suboptimal pulmonary vessel growth during the post-Norwood stage of shunt-dependent pulmonary flow, mainly in the case of hypoplastic left heart syndrome.

### Results from Other Volume Expansion Studies

De May et al. [5] performed RVE as part of routine diagnostic heart catheterization in 28 patients during 32 procedures. Despite measuring the venous, pulmonary arterial, and ventricular pressures, their focus was on the precapillary pressure response and underlying transpulmonary flow reserve.

Averin et al. [6] also included rapid saline infusion into routine catheterization of all Fontan patients during a certain period. They measured both CVP and VEDP in 46 patients and, in their retrospective study, demonstrated occult diastolic dysfunction in 35% of patients. However, no imaging data were acquired, which would have permitted discrimination between systolic and diastolic dysfunction, leading to pre-cardiac pressure rise.

While pulmonary vascular resistance has been the focus of non-invasive interventions, our data suggest the equal importance of impaired ventricular function for long-term function of Fontan circulation. Hence, it will be of interest if the pressure rise in the preload-stressed Fontan circulation corresponds with the directly measurable limitations of myocardial contractile reserve.

### Limitations

Our study design did not include some known factors that influence hemodynamics and/or CRF in patients with Fontan circulation, including veno-venous collaterals [22], echocardiographic signs of ventricular dysfunction at rest [23], atrioventricular valve incompetency [24], chronotropic incompetency [25], and differences in energy loss in the Fontan pathway [26].

The pressure difference CVP‒VEDP does not equal the transpulmonary pressure gradient, which must be calculated from simultaneously measured pre- and post-capillary mean pressures; our experimental position of catheters did not allow such measurement.

## Conclusions

Preload challenge by rapid saline infusion unmasked occult limitations of Fontan circulation indicated by the elevation of both CVP and VEDP. Baseline abnormality and volume-induced elevation of both CVP and VEDP were associated with impaired CRF. Transpulmonary flow limitation was more pronounced in RV morphology. Our findings suggest that ventricular dysfunction might play an important role in functional impairment after Fontan operation in young adults as pulmonary vascular resistance.

## Data Availability

Anonymized study data will be provided on reasonable request to the corresponding author.
